# A robust scoring system to evaluate sepsis severity in an animal model

**DOI:** 10.1186/1756-0500-7-233

**Published:** 2014-04-12

**Authors:** Bradly Shrum, Ram V Anantha, Stacey X Xu, Marisa Donnelly, SM Mansour Haeryfar, John K McCormick, Tina Mele

**Affiliations:** 1 Division of General Surgery, Department of Surgery, Schulich School of Medicine and Dentistry, Western University, London, Ontario, Canada; 2 Department of Microbiology and Immunology, Schulich School of Medicine and Dentistry, Western University, London, Ontario, Canada; 3 Division of Critical Care, Department of Medicine, Schulich School of Medicine and Dentistry, Western University, London, Ontario, Canada; 4 Division of General Surgery and Critical Care, Room A8-124, London Health Sciences Centre-University Hospital, 339 Windermere Road, London, ON N6A 5A5, Canada

**Keywords:** Sepsis score, Sepsis, Septic shock, Peritonitis, Animal model, Cytokine analysis, Infectious diseases

## Abstract

**Background:**

The lack of a reliable scoring system that predicts the development of septic shock and death precludes comparison of disease and/or treatment outcomes in animal models of sepsis. We developed a murine sepsis score (MSS) that evaluates seven clinical variables, and sought to assess its validity and reliability in an experimental mouse model of polymicrobial sepsis.

**Methods:**

Stool collected from the cecum of C57BL/6 (B6) mice was dissolved in 0.9% normal saline (NS) and filtered, resulting in a fecal solution (FS) which was injected intraperitoneally into B6 mice. Disease severity was monitored by MSS during the experimental timeline. Blood and tissue samples were harvested for the evaluation of inflammatory changes after sepsis induction. The correlation between pro-inflammatory markers and MSS was assessed by the Spearman rank correlation coefficient.

**Results:**

Mice injected with FS at a concentration of 90 mg/mL developed polymicrobial sepsis with a 75% mortality rate at 24 hours. The MSS was highly predictive of sepsis progression and mortality, with excellent discriminatory power, high internal consistency (Cronbach alpha coefficient = 0.92), and excellent inter-rater reliability (intra-class coefficient = 0.96). An MSS of 3 had a specificity of 100% for predicting onset of septic shock and death within 24 hours. Hepatic dysfunction and systemic pro-inflammatory responses were confirmed by biochemical and cytokine analyses where the latter correlated well with the MSS. Significant bacterial dissemination was noted in multiple organs. Furthermore, the liver, spleen, and intestine demonstrated histopathological evidence of injury.

**Conclusions:**

The MSS reliably predicts disease progression and mortality in an animal model of polymicrobial sepsis. More importantly, it may be used to assess and compare outcomes among various experimental models of sepsis, and serve as an ethically acceptable alternative to death as an endpoint.

## Background

Sepsis remains one of the leading causes of death in non-coronary intensive care units (ICUs) [1,2], with a mortality rate of approximately 30% despite optimal therapeutic intervention. Moreover, the mortality rate rises up to 70% [2,3] when there is ongoing organ dysfunction or severe sepsis [4]. Close to two million cases of sepsis are identified annually worldwide, although this figure may be a gross underestimation because of inadequate diagnosis [3]. Defined as an overwhelming and dysregulated inflammatory response that is often initiated by infection, sepsis disrupts the cardiovascular, immunological, and endocrine systems in a complex, patient- and organism-specific manner, making it difficult to study this disease clinically. Animal models that attempt to mimic the complexities of sepsis in the laboratory setting have yielded new insights into the pathophysiology and molecular mechanisms of this illness. However, the failure of therapies that showed promise during the preclinical stages, but yielded little benefit in human trials [5,6], have led to renewed calls for a closer examination into the shortcomings of conventional animal models of sepsis [5,7].

Conventional animal models of sepsis are typically microbial-based responses which may be mono- or poly-microbial in design. Using a single organism results in a septic state that is dependent on the type and amount used as well as the animal and route of administration [8], whereas a polymicrobial model typically mimics intra-abdominal sepsis, which affects 20% of patients admitted with sepsis [9]. Polymicrobial models of sepsis typically involve either fecal-induced peritonitis (FIP) or cecal ligation and puncture (CLP). In the FIP model, a freshly prepared fecal solution is derived from murine colonic stool and injected intraperitoneally into mice at a defined concentration to result in acute peritonitis. In the CLP model, the cecum is ligated in a non-obstructing manner, and punctured to allow fecal content to leak into the normally sterile peritoneal cavity [10]; a mixed bacterial infection with an inflammatory source of necrotic intestinal tissue is subsequently established [11-13]. Irrespective of the model used to induce and evaluate sepsis, however, the paucity of reliable validated scoring systems that can be applied to animals severely limits comparison of disease outcomes and treatment regimens across different experimental models of sepsis. Given that sophisticated scoring systems, such as the Acute Physiology and Chronic Health Evaluation (APACHE) II, Simplified Acute Physiology Score (SAPS) II, and Sequential Organ Failure Assessment (SOFA) score, can evaluate the degree of critical illness and provide prognostic clarity for patients with sepsis [14], the development of similar metrics for animal models would allow for the consistent and standardized reporting of disease severity and therapeutic outcomes. A pioneering study by Huet and colleagues [15] demonstrated that a mouse clinical assessment score for sepsis (M-CASS) provided a consistent and objective evaluation of disease severity in a mouse model of *Klebsiella pneumonia*-induced pneumonia, with high reliability and reproducibility. Eight criteria (fur aspect, activity, posture, behaviour, respiration, chest sounds, eyes, and body weight) were assessed to provide an overall score between 1 and 4, which subsequently dictated the frequency of further monitoring and/or euthanasia. In our present study, we independently developed and validated a murine sepsis score (MSS) which evaluates seven individual criteria, and results in an aggregate score to evaluate the clinical condition of mice with experimental polymicrobial sepsis. Furthermore, we assessed the specificity and sensitivity of the MSS for predicting the onset of severe sepsis and death in a mouse model of FIP, and evaluated its reproducibility and reliability in corroborating with sepsis severity.

## Materials and methods

### Animals

Male C57BL/6 mice, 10–12 weeks of age, were purchased from Charles River Laboratories International, Inc. (Wilmington, MA) and placed in standard housing in the Health Sciences Animal Care Facility at Western University (London, ON, Canada). Animals had an average weight of 22.5 g (range, 21–25 g) prior to the start of the experiments. Animal husbandry conditions included a room temperature of 23°C, humidity of 50%, and a 12-hour light–dark cycle (dark from 1900 h to 0700 h). Bedding in cages consisted of sawdust and wood shavings, while corn mash and water (in a stoppered-bottle with a nose-activated nozzle) was available for mice to feed *ad lib*. Cages also contained an igloo to allow nesting. Animals were housed with one to three cage mates. All animal procedures were done in accordance with guidelines set by the Canadian Council on Animal Care and approval was granted by the Western University Animal Use Subcommittee (no. 2008–034).

### Sepsis model

#### Preparation of fecal slurry

The solution used to cause FIP was made by the following procedure: fresh feces were collected from the lower cecum of euthanized donor mice, weighed, and mixed with a calculated volume of saline solution to give fecal concentrations of 45, 90, and 180 mg/mL. To ensure reproducibility, the procedure was standardized by the use of fresh solution prepared from mice living in the same conditions as the experimental animals. The solution was pressed through a 70-μm nylon mesh strainer (BD Biosciences, Franklin, NJ) to remove particulate matter.

#### Induction of sepsis

For sepsis induction, each mouse was given an intraperitoneal (i.p.) injection of 0.5 mL of the fecal slurry solution using a syringe and 27G needle. Depending on the fecal concentrations (45, 90, and 180 mg/mL) used, each mouse therefore received 2, 4, or 8 mg of FIP solution per 1 g body weight respectively. Sham mice were injected with sterile normal saline (NS). Pain (either from the injection or from the fecal slurry)was assessed using facial expression as described by Langford *et al.*[16], as well as body posture and vocalization. Analgesia was provided by a subcutaneous injection of buprenorphine (0.1 mg/kg).

#### Monitoring of mice

Monitoring of the health of the animals was conducted by two investigators every 2 hours after the induction of sepsis for 12 hours, and then every hour thereafter: one of the investigators was blinded to the treatment so as to test for the reproducibility of the MSS. Mice were evaluated while they were still in their cages (with the lids removed for better visualization).

### Development of the MSS

In a pilot study involving thirty mice that were given 90 mg/mL fecal slurry and observed over 24 hours, a veterinarian from the Animal Care and Veterinary Services (ACVS, Western University) and the primary author of this study (Shrum) assessed the mice jointly using variables that have been described in the literature [8,15,16]. Certain variables such as temperature and weight loss did not change during the experimental timeline, while fewer than 5 % of mice needed analgesia for pain immediately after the fecal slurry injection. Consequently, the final variables that were incorporated into the MSS (Table 1) included spontaneous activity, response to touch and auditory stimuli, posture, respiration rate and quality (laboured breathing or gasping), and appearance (i.e. degree of piloerection). Each of these variables are given a score between 0 and 4, and are detailed in Table 1. Mice were euthanized if the MSS at any given time point was greater than 21, or if the points ascribed to respiratory rate or quality increased by more than 3.

**Table 1:** Murine Sepsis Score (MSS) to assess the severity of disease in an experimental model of fecal-induced peritonitis

**Variable**	**Score and description**
Appearance	0- Coat is smooth
	1- Patches of hair piloerected
	2- Majority of back is piloerected
	3- Piloerection may or may not be present, mouse appears “puffy”
	4- Piloerection may or may not be present, mouse appears emaciated
Level of consciousness	0- Mouse is active
	1- Mouse is active but avoids standing upright
	2- Mouse activity is noticeably slowed. The mouse is still ambulant.
	3- Activity is impaired. Mouse only moves when provoked, movements have a tremor
	4- Activity severely impaired. Mouse remains stationary when provoked, with possible tremor
Activity	0- Normal amount of activity. Mouse is any of: eating, drinking, climbing, running, fighting
	1- Slightly suppressed activity. Mouse is moving around bottom of cage
	2- Suppressed activity. Mouse is stationary with occasional investigative movements
	3- No activity. Mouse is stationary
	4- No activity. Mouse experiencing tremors, particularly in the hind legs
Response to stimulus	0- Mouse responds immediately to auditory stimulus or touch
	1- Slow or no response to auditory stimulus; strong response to touch (moves to escape)
	2- No response to auditory stimulus; moderate response to touch (moves a few steps)
	3- No response to auditory stimulus; mild response to touch (no locomotion)
	4- No response to auditory stimulus. Little or no response to touch. Cannot right itself if pushed over
Eyes	0- Open
	1- Eyes not fully open, possibly with secretions
	2- Eyes at least half closed, possibly with secretions
	3- Eyes half closed or more, possibly with secretions
	4- Eyes closed or milky
Respiration rate	0- Normal, rapid mouse respiration
	1- Slightly decreased respiration (rate not quantifiable by eye)
	2- Moderately reduced respiration (rate at the upper range of quantifying by eye)
	3- Severely reduced respiration (rate easily countable by eye, 0.5 s between breaths)
	4- Extremely reduced respiration (>1 s between breaths)
Respiration quality	0- Normal
	1- Brief periods of laboured breathing
	2- Laboured, no gasping
	3- Laboured with intermittent gasps
	4- Gasping

#### Euthanasia of mice

At the conclusion of the experiments, animals were sacrificed and post-mortem laparotomy was performed in order to collect tissues. Mice were anesthetized with 100 mg/kg ketamine (Bioniche Life Sciences, Belleville, ON) and 5 mg/kg xylazine (Bayer AG, Leverkusen, Germany), and euthanized by cardiac puncture using a 27G needle and 3-mL syringe.

### Blood and tissue collection and CFU determination

Blood collected from the cardiac puncture of mice during euthanasia was centrifuged to separate the serum, which was stored at -80°C until use for biochemistry assays and cytokine analysis. The liver, spleen, kidneys, heart, lungs, lower intestine and brain were fixed in buffered 4% formalin, embedded in paraffin, and used for hematoxylin and eosin (H&E) staining. In separate experiments, tissues were collected for homogenization and plating on brain heart infusion (BHI) agar media for overnight growth in an aerobic chamber at 37°C to determine the number of bacterial colony-forming units (CFUs) per organ [17].

### Histopathology

Tissues were harvested and fixed using 4% formaldehyde. Tissues were then embedded using paraffin and serially sectioned (4-μm) *in toto*. Every third slide was stained with H&E for histopathological analysis. Two images per slide were captured with a microscope (Motic B1; Houston, TX) at a magnification of 20× and at a resolution of 1098 × 768 pixels. A pathologist (Dr. Aaron Haig, Department of Pathology, Western University), who was blinded to the experimental conditions, examined the tissue sections.

### Terminal deoxynucleotidyltransferasedUTP nick end labelling (TUNEL) assay

This procedure was performed as described previously [18]. Briefly, 4-μm sections were deparaffinized in xylene, rehydrated in graded alcohol, and rinsed in distilled water. Antigen unmasking was accomplished using freshly prepared Proteinase K solution (10 μg/mL) for 60 min at 37°C. After washing twice with distilled water, sections were incubated with TdT enzyme (75 U/mL; EMD Millipore Corp., Billarica, MA) and digoxigenin-11-UTP (5 nmol) for 90 min at 37°C. The slides were then washed in SSC buffer (150 mmolNaCL, 15 mmol sodium citrate, pH 7.0), followed by Tris–HCl buffer (10 mmolTris, 150 mmolNaCl, pH 8.2) for 1 min per wash. A blocking agent was used to prevent non-specific binding, and sections were developed with a Fab fragment against digoxigenin linked to alkaline phosphatase and fast red chromogen. Sections were then washed and counter stained.

### Serum biochemistry assays

Serum levels of alanine aminotransferase (ALT), aspartate aminotransferase (AST), and lipase, expressed as U/L, as well as creatinine (expressed as mg/dL), glucose (mg/dL), and albumin (mg/dL) were measured using a commercially available diagnostic kit (Catachem Inc., Oxford, CT) according to the manufacturer’s instructions.

### Multiplex cytokine array

Serum was analyzed by bead-based multiplex assay for 32 different cytokines, chemokines, and growth factors (Eve Technologies, Calgary, Alberta, Canada) including granulocyte-colony stimulating factor (G-CSF), granulocyte macrophage-colony stimulating factor (GM-CSF), interleukin (IL)-1α, IL-1β, IL-3, IL-4, IL-5, IL-6, IL-7, IL-10, IL-12 (p40), IL-12 (p70), IL-13, IL-15, IL-17A, IP-10, keratinocyte chemo-attractant (KC), leukemia inhibitory factor (LIF), monocyte chemotactic protein (MCP)-1, monocyte-colony stimulating factor (M-CSF), monokine induced by gamma interferon (MIG), macrophage inflammatory protein (MIP)-1α, MIP-1β, MIP-2, Regulated on Activation, Normal T cell Expressed and Secreted (RANTES), tumour necrosis factor (TNF)-α, and vascular endothelial growth factor (VEGF).

### Statistical analysis

Data are expressed as mean and SEM. For the MSS, serum biochemistry, and multiplex cytokine assay, data from sham animals obtained at all the time points were pooled and then employed as a common control group as Friedman’s test indicated no differences over time. Data obtained at indicated time points from septic animals were considered independently. Consequently, differences between groups were analyzed by applying one-way analysis of variance (ANOVA) followed by Dunn’s pairwise comparison for post-hoc analysis. For statistical testing of all other results, differences between groups were analyzed by ANOVA or Mann–Whitney test. Group sizes reported for biochemistry data varied over time, reflecting the mortality rate in septic animals. Logarithmic transformation of bacterial counts was performed for analysis of bacterial burden in tissues. In all analyses, two-tailed *p* values less than 0.05 were considered statistically significant.

Each of the seven variables measured as part of the MSS consists of five possible scores (0 to 4). The internal consistency of the MSS and each of the variables was assessed by Cronbach’s alpha. Inter-rater reliability of the MSS was also assessed by calculating the intraclass coefficient (ICC), comparing each assessor’s independent scores for each mouse (sham and septic) at 2, 12, 14, 16, 18, 20, and 24 hours. Additionally, the ability of the MSS to discriminate between sham and septic mice was tested using the receiver operating characteristic (ROC) curve, and by quantifying the area under the curve (AUC) [19]. An AUC between 0.7 and 0.8 is classified as “acceptable,” and an AUC between 0.8 and 0.9 is considered to have an “excellent” discrimination [19]. For the MSS, the score giving the best Youden index was determined to be the cutoff point [20]: the sensitivity, specificity, and positive and negative predictive values were calculated based on this score.

To ensure that the MSS reflected the severity of the septic insult, correlations between the sepsis score and serum pro-inflammatory cytokine levels were performed by calculating the Spearman rank correlation coefficient (Spearman’s rho). All data analysis was performed using Graphpad Prism Version 5.01 (Graphpad, La Jolla, California).

## Results

**Figure 1:**
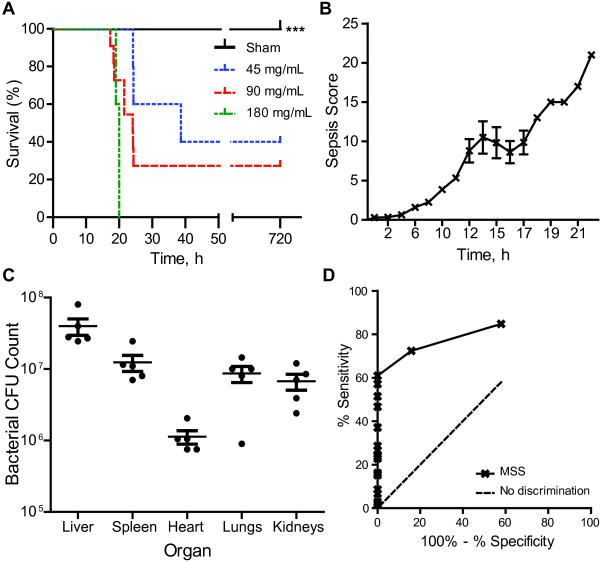
**Characterization of the FIP sepsis model in mice. (A)** Mouse survival over time versus concentration of fecal solution (FS; *n* = 20 mice per concentration of FS). **(B)** Murine Sepsis Score (MSS) over time of mice that were administered 90 mg/mL FS (*n* = 20, 1 representative experiment). **(C)** Viable bacterial colony forming units (CFU) recovered from solid organs of mice treated with 90 mg/mL FS, at the time of euthanasia (*n* = 5). **(D)** Receiver-operator curve (ROC) evaluating the sensitivity and specificity of the MSS in differentiating healthy mice from those that progress to septic shock and death.

A total of 300 mice were used in the survival experiments that were conducted independently over a period of two years. Compared to sham-treated mice (*n* = 60), which had a survival of 100% throughout the experimental timeline, FIP mice had a survival rate of 0% for 180 mg/mL FS (*n* = 20), 25% for 90 mg/mL FS (*n* = 200) and 40% for 45 mg/mL FS (*n* = 20) at 24 hours post-FIP induction (Figure 1A). For subsequent experiments, we used a FS concentration of 90 mg/mL to mimic the clinical mortality of 70-80% in severe, untreated intra-abdominal sepsis [2,3].

The MSS for FIP mice at a concentration of 90 mg/mL are shown in Figure 1B. Compared to sham-treated mice with a mean score of 1 after 24 hours, FIP-treated mice had significantly higher (*p* < 0.0001) sepsis scores. Between 0 to 11 hours post-FIP induction, mouse scores remained relatively consistent as assessed by independent observers, with mild piloerection and decreased movement. After 12 hours, septic mice appeared to progressively manifest additional symptoms including decreased respiratory rate, increasingly laboured breathing and minimal response to auditory and tactile stimuli. Between 12 to 17 hours post-FIP induction, variability in sepsis severity scores was observed to be due to differences in respiratory rates and quality of breathing as well as response to tactile and auditory stimuli. The intra-class correlation coefficient for comparison between the blinded and non-blinded assessors of septic mice was 0.96 (95% CI: 0.92 - 0.98), indicating excellent inter-rater reliability. The Cronbach alpha coefficient was 0.92, indicating excellent internal consistency of the MSS.

For a concentration of 90 mg/mL FS, we calculated a mortality rate of 42% within 1 hour of attaining a score of 10, and a mortality of 75% within 2 hours of attaining a score of 10. Fifty-seven percent of mice that reached a score of 15 died or had to be euthanized (in accordance with guidelines set by the Animal Use Subcommittee) within 1 hour, and 86% of mice that reached a score of 15 died within 2 hours. Based on the ROC curve generated for the MSS (Figure 1D), the AUC (95% confidence interval) was 0.825 (0.752 - 0.898) with a *p* value < 0.0001, suggesting that the scoring system has excellent discriminatory power. A MSS of 3 (Youden score of 0.61) was selected as the cut-off point for mice that progressed to severe sepsis post-FIP induction: the sensitivity (±95% C.I.) and specificity (±95% C.I.) of this score was 57% (47-67%) and 100% (82-100%), respectively.

When organs were homogenized and plated on agar, bacterial growth was observed in all tissues, including liver, spleen, heart, lung, and kidneys (Figure 1C). Consistent with the polymicrobial nature of the model, significant variations in colony size (ranging from 1–3 mm in diameter), colour (white, brown, and yellow), and CFU counts were observed. Bacterial counts were not observed in any organs recovered from sham mice (data not shown). We did not observe a correlation between sepsis score and CFU counts in FIP mice.

On necropsy, FIP mice were observed to have developed diffuse intestinal distension compared to uninfected control mice (Additional file 1: Figure S1). In addition, FIP mice had peritoneal and mesenteric lymphadenopathy, and rarely developed abscesses. We also routinely observed the presence of a yellow fibrin film on the surfaces of the intra-abdominal organs, most notably overlying the liver and spleen. We did not identify any grossly visible areas of necrosis or ischemia within the organs. In mice that were euthanized due to severe respiratory distress, we observed minor pulmonary haemorrhage and the lungs appeared edematous.

**Figure 2:**
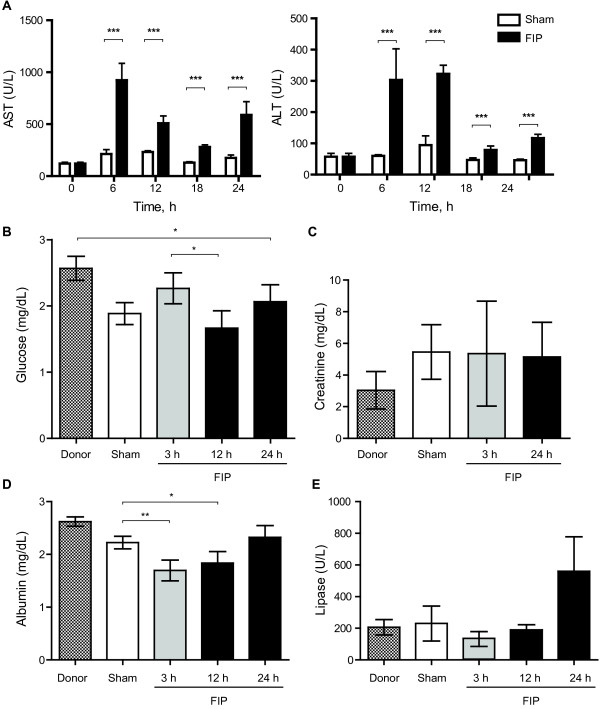
**Blood serum biochemistry of (A) liver enzymes aspartate transaminase (AST) and alanine aminotransferase (ALT), (B) Glucose, (C) Creatinine, (D) Albumin, and (E) Lipase (*n*** **= 4 in donor group;*n*** **= 12 for sham group;*n*** **≥ 4 per group at 3 h, 12 h, and 24 h).** Mean values shown with SEM error bars. *p < 0.05, **p < 0.01, ***p < 0.001.

Serum biochemistry demonstrated significantly elevated AST and ALT levels in the FIP group at 6, 12, 18, and 24 hours post-sepsis induction (*p* < 0.001) compared to the sham group (Figure 2A). However, both AST and ALT levels peaked at 6 to 12 hours in septic mice: while the AST levels declined and rose again at 18 and 24 hours respectively, the ALT levels fell sharply at 18 and 24 hours. A linear correlation between liver transaminases and MSS was only significant for the first 12 hours of the experimental timeline, but was non-significant for the entire duration (24 hours) of the experiment. Serum glucose and creatinine did not demonstrate significant changes over time in the FIP group (Figure 2B and C). Serum albumin levels decreased significantly at 3 and 12 hours post-sepsis compared to the sham group (*p* = 0.0057 and *p* = 0.018, respectively), but there was no difference in albumin levels after 24 hours (Figure 2D). We observed a trend towards higher lipase levels at 24 hours post-sepsis but there was significant variability in lipase activity among individual mice in the FIP group (Figure 2E).

**Figure 3:**
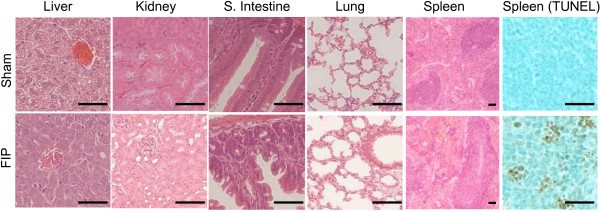
Histology of representative tissues from control and FIP (90 mg/mL) at 24 h developed with TUNEL or haematoxylin and eosin (size bar, 50 μm).

Histological examination of the tissues demonstrated different degrees of pathology in various organs at 24 hours after FIP (Figure 3). In the lung, we observed mild edema in the alveolar spaces and leukocyte accumulation in the peripheries of pulmonary arterioles. In the liver of mice with FIP, parenchymal cells demonstrated vacuolization, limited necrosis, and loss of organization and structure. We also occasionally observed capsular edema and recruitment of inflammatory cells onto the liver surface. The spleen demonstrated significant changes post-sepsis, with expansion of the white pulp, and widespread cellular apoptosis, which was also confirmed by TUNEL staining (Figure 3). At higher concentrations of fecal slurry (180 mg/mL), pathological changes associated with damage and inflammation could be observed within 12 hours of insult (data not shown). We also observed pathological changes in the small intestine (Figure 3), characterized by the loss of goblet cells and loss of villi. We did not observe the accumulation of neutrophils or other leukocytes within the submucosa, but we occasionally observed necrosis and debris on the serosal surfaces of the gastrointestinal tract. We did not observe pathological changes in the hearts or brains of septic mice at 24 hours (data not shown).

**Figure 4:**
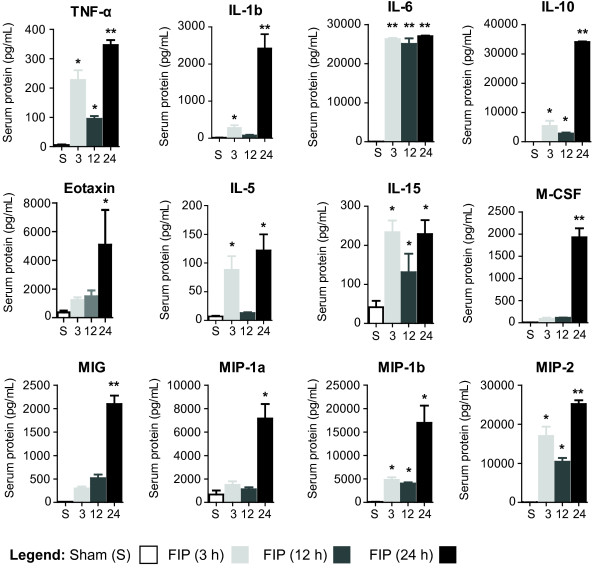
**Blood serum cytokine and chemokines.** Sham and FIP (90 mg/mL) cytokine and chemokine levels (pg/mL) at 3, 12, and 24 h post FIP induction. Mean serum protein concentrations ± SEM are shown (*n* = 12 for sham group; *n* ≥ 3 for 3, 12, and 24 h groups). *p < 0.05, **p < 0.01, ***p < 0.001.

Analysis of cytokine levels by multiplex array showed a rapid, sustained, and significant increase of the putative markers of experimental sepsis, namely IL-1β, IL-6, IL-10, and TNF-α, in FIP mice over a 24-hour period (*p* < 0.001) versus the sham group (Figure 4). Additionally, we observed increased levels of eotaxin, M-CSF, MIG, MIP-1α, MIP-1β, MIP2, IL-5 and IL-15. IL-5 and IL-15 returned to baseline levels by 12 hours; however, IL-5 was detected at significantly increased levels at 24 hours, compared to the control group (*p* < 0.001). Results of additional analysed cytokines, which are well described in septic models, are shown in Additional file 2: Table S1.We also performed a correlation analysis between serum cytokine levels and the MSS. The levels of IL-6 (Spearman’s rho = 1.0, p = 0.042), eotaxin (Spearman’s rho = 1.0, p = 0.042), G-CSF (Spearman’s rho = 1.0, p = 0.042), M-CSF (Spearman’s rho = 1.0, p = 0.042), MIG (Spearman’s rho = 1.0, p = 0.042), and RANTES (Spearman’s rho = 1.0, p = 0.042) demonstrated significant correlations with MSS throughout the experimental timeline.

## Discussion

The development of a reliable, sensitive, and specific scoring system can effectively facilitate comparison of disease outcomes and therapeutic regimens [5,7,21] across multiple models of sepsis, and improve the clinical relevance of novel diagnostic biomarkers [21]. In this study, we demonstrate a robust and comprehensive scoring system with high internal consistency, excellent inter-rater reliability, and high specificity and sensitivity in predicting severe sepsis and mortality during the experimental timeline.

The MSS reliably predicts 1-hour and 2-hour mortality when clinical scores are greater than 10, with excellent discriminatory capacity. Mice that attained a clinical score of three had 100% specificity for dying from sepsis during the experimental timeline. Our scoring system uses easily observed mouse behaviours and changes in appearance that have been identified as important factors in measuring septic morbidity [8]. By identifying predictable changes in appearance, activity, and responses to stimuli in our FIP mice, we were able to translate our observations into a scoring system that could be used to monitor and evaluate sepsis severity in our animal model.

Few studies have characterized the development of a sepsis score that can reliably predict mortality in animal models. Huet and colleagues developed a novel “Mouse Clinical Assessment Score for Sepsis” (M-CASS) for use in a mouse model of pneumonia [15]. In the M-CASS scoring approach, a score between 1 and 4 was given after evaluation of variables such as the degree of piloerection, activity, posture, behaviour, chest movements, chest sounds, eye opening, and weight loss. Whereas the M-CASS provides one score that encompasses all eight variables, the scoring system in our study provides an individual score for each variable, which can then be added together for an overall MSS. Regardless of the approach used, both the M-CASS and MSS demonstrate high reliability and internal consistency, as well as excellent inter-observer reliability in evaluating septic animals. Moreover, the findings of our study corroborate the observations of Huet *et al.*, suggesting that a clinical scoring system offers an effective surrogate end point that is more ethically acceptable than death [8,15].

Because the MSS is based on simple observation, it may be easily implemented in any laboratory that utilizes a mouse model of sepsis. Moreover, it is also fully compatible with any investigative timeline because of its non-invasive nature. Other methods to evaluate sepsis severity in mouse models, including arterial pressure monitoring, serum biochemistry assays, and cytokine arrays can be difficult to perform in small animals. In addition, only a limited amount of blood can be sampled from septic mice without compromising the health of the animal, necessitating the sacrifice of a large number of animals to perform adequate analyses. Death, however, is not an acceptable endpoint for animal models of sepsis at many institutions [8]; therefore, investigators must carefully design experiments to balance the accurate collection of data while minimizing animal suffering [5,6,8,15]. Since the criteria for euthanasia may vary widely and depend on the subjective evaluation by the investigator, adoption of our scoring system will provide a uniform approach to addressing criteria for euthanasia. The MSS may also be used in microbe-specific models of sepsis, although calibration of the scoring system will be necessary to optimize sensitivity and specificity. This would be especially important for models that require post-surgical recovery from anaesthesia [12,13], which may confound early measures of activity and level of consciousness. As animal models continue to be refined in order to more closely mimic the human condition, the MSS may remain effective and applicable because of its emphasis on clinical characteristics.

We elected to use the FIP model to test the validity of the MSS because it was easy to standardize and conduct in a controlled setting, given that multiple operators with varying levels of surgical expertise were conducting the experiments in three different laboratories. Outcomes in our model could be changed simply by varying the concentration of feces in solution, unlike the CLP model, which requires changes to the surgical protocol (such as needle size, number of punctures, and manual extrusion of cecal contents) to alter disease outcomes [10]. The need for surgical anesthesia is also obviated with the FIP model, removing another confounding factor that can affect animal behaviour in the immediate post-surgical period [22]. Ensuring that our FIP model was successfully mimicking the pro-inflammatory response seen in clinical sepsis [23], we consistently demonstrated bacterial growth in every major organ in septic mice, with similar tissue CFUs in independently performed experiments. While this study was limited by the inability to culture and identify strictly anaerobic microorganisms, we observed colonies that varied in size, shape, and color, confirming the polymicrobial nature of the infection. The biochemistry data, with the clear evidence of progressive hepatic dysfunction and failure, and the overwhelming cytokine dysfunction, likely contributed to the morbidity and mortality of our mice.

The characteristic cytokine markers of sepsis [24,25], including IL-1β, IL-6, IL-10 and TNF-α, increased significantly during the experimental timeline. For what we believe is the first time, however, we also demonstrate that levels of eotaxin, IL-5, IL-15, M-CSF, MIG, MIP-1α, MIP-1β, and MIP-2 rise significantly during sepsis. Eotaxin is associated with eosinophil recruitment and function [26], and also inhibits neutrophil recruitment during the acute inflammatory phase of sepsis. IL-15, M-CSF, MIG, MIP-1α, MIP-1β and MIP-2 promote cellular differentiation, activity, survival, recruitment and chemotaxis [27-29], confirming the complexity of the dysregulated immune response during sepsis. In addition, the levels of certain pro-inflammatory cytokines (eotaxin, IL-6, G-CSF, M-CSF, MIG, and RANTES) correlate strongly with the MSS, confirming that the score reflects the severity of the septic insult. Some pro-inflammatory markers such as TNF-α and IL-1β did not demonstrate correlation with the MSS, although they were significantly elevated in septic mice compared to sham-treated mice; this may have been due to the early elevation of these markers during the experimental timeline, or due to the variability from pooling cytokine data from multiple independently-performed experiments.

Interestingly, the histological changes we noted in organs from septic mice were subtle despite the obvious clinical severity of the animals, an observation that correlates with other studies [30,31] utilizing both FIP and CLP models. In their CLP model, for example, Doi *et al.* observed renal tubular damage mainly consisting of tubular vacuolization [32,33] but no changes in the lung, liver, or intestinal tract [31]; Fisher *et al.* did not observe significant changes in the lungs or kidneys of FIP mice [30]. Despite the significant intestinal distension seen consistently in septic mice within our study, histology of the gastrointestinal tract manifested subtle changes that have been described in other sepsis models, including the loss of glandular structure and intestinal epithelial villi, edema of the lamina propria, capillary hemorrhage, ulceration and apoptosis [34]. Apoptosis was also most evident in the spleen, as confirmed by TUNEL staining, and likely involves the lymphocytes, as has been previously shown in the CLP model by Hotchkiss *et al.*[35,36]. These results corroborate human studies indicating that apoptotic factors may be early biomarkers of sepsis that modulate lymphocyte and monocyte activity [23,36]. In the lungs, we observed minimal extravasation of red cells,but little accumulation of inflammatory cells into the air spaces as described by Zingarelli *et al.*[37].

## Conclusion

In conclusion, we present a validated MSS that can be applied reliably, consistently, and independently in an animal model of sepsis, with high specificity and sensitivity for predicting the onset of severe sepsis and death during the experimental timeline. Utilization of this scoring system may not be limited to the fecal-induced peritonitis model used in this study, and may allow for a standardized method of describing disease outcomes across various experimental animal models of sepsis. Most importantly, the assessment of clinical status in septic animals may be an ethically acceptable alternative to death as an endpoint in animal models of sepsis.

## Competing interests

The authors do not have any actual or potential conflicts of interest or competing financial interests to declare.

## Author contributions

BS and RVA designed and conducted the experiments, performed data analysis, and drafted the manuscript. SXX and MD conducted experiments, and assisted with data analysis. SXX, SMMH, JKM, and TM provided critical revisions of the manuscript for important intellectual content. All authors read and approved the final version of this manuscript.

## Supplementary Material

Additional file 1: Figure S1 Macroscopic intra-abdominal view of control (*left*) and 90 mg/mL FIP mouse (*right*) at 24 h reveals significant intestinal distension in the latter (size bar, 1 cm). Click here for file

Additional file 2: Table S1 Changes in concentrations of chemokines and cytokines in sham- and 90 mg/mL FS-treated mice with FIP. Click here for file
