# Supplementary material for: A robust scoring system to evaluate sepsis severity in an animal model
Source: BMC Res Notes. 2014 Apr 12;7:233. doi: 10.1186/1756-0500-7-233 (PMC4022086; doi:10.1186/1756-0500-7-233)
Supplement: Additional file 1: Figure S1 — Macroscopic intra-abdominal view of control (left) and 90 mg/mL FIP mouse (right) at 24 h reveals significant intestinal distension in the latter (size bar, 1 cm). [file 1756-0500-7-233-S1.docx]

# Supplementary Figure S1


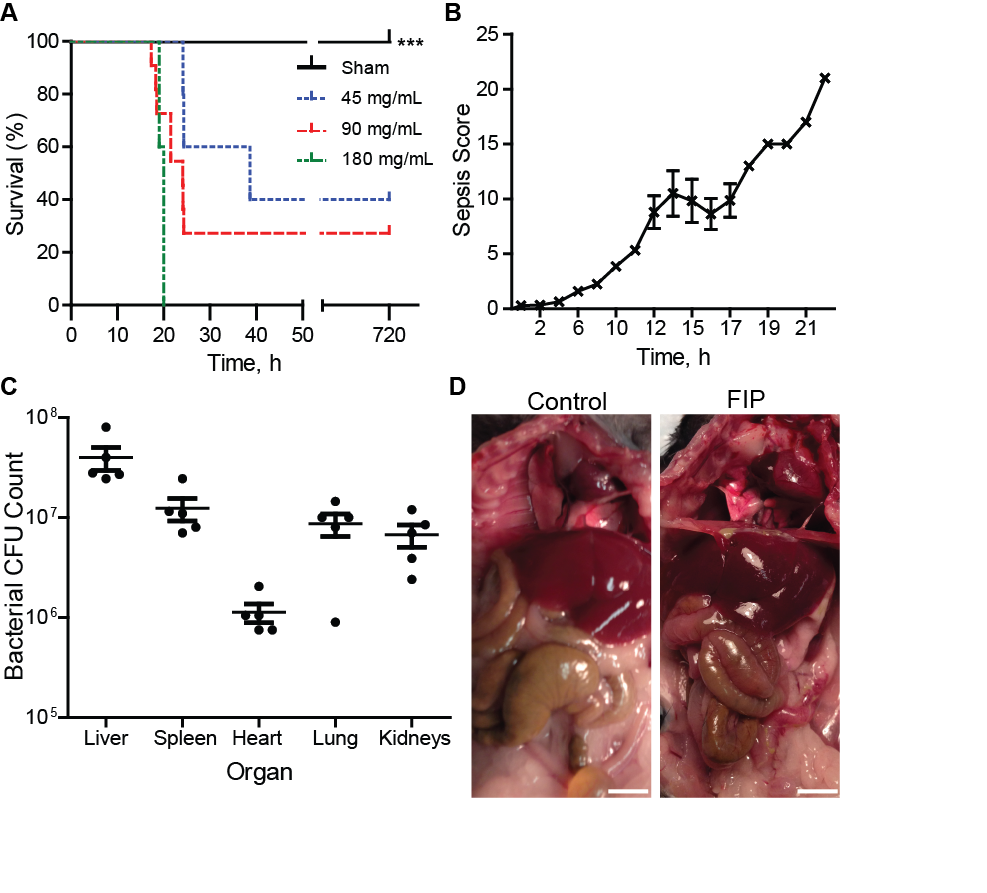


**Supplementary Figure S1:** Macroscopic intra-abdominal view of control (*left*) and 90mg/mL FIP mouse (*right*) at 24h reveals significant intestinal distension in the latter (size bar, 1 cm).
