# Supplementary material for: A robust scoring system to evaluate sepsis severity in an animal model
Source: BMC Res Notes. 2014 Apr 12;7:233. doi: 10.1186/1756-0500-7-233 (PMC4022086; doi:10.1186/1756-0500-7-233)
Supplement: Additional file 2: Table S1 — Changes in concentrations of chemokines and cytokines in sham- and 90 mg/mL FS-treated mice with FIP. [file 1756-0500-7-233-S2.docx]

**Supplementary Table S1**: Changes in concentrations of chemokines and cytokines in sham- and 90 mg/mL FS-treated mice with FIP.

| Cytokine / Chemokine | Sham | FIP | | | P Value |
| --- | --- | --- | --- | --- | --- |
|  |  | 3h | 12h | 24h |  |
| IL-1α | 81.14 ± 22.79 | 313.91 ± 25.18 | ND | 1089.33 ± 141.89 | <0.001 |
| IL-12p40 | 10.14 ± 4.10 | 66.01 ± 13.74 | 0.91 ± 0.91 | 86.76 ± 8.97 | <0.001 |
| IL-12p70 | 39.81 ± 24.53 | 48.14 ± 17.62 | 28.78 ± 17.15 | 70.83 ± 36.10 | ns |
| IL-13 | 218.93 ± 31.44 | 413.71 ± 17.57 | 315.23 ± 39.11 | 812.51 ± 171.84 | <0.001 |
| IL-17A | 5.45 ± 1.13 | 403.32 ± 132.46 | 103.32 ± 33.20 | 1954.34 ± 538.63 | <0.001 |
| IP-10 | 59.42 ± 9.45 | 553.66 ± 223.84 | 155.26 ± 51.19 | 1059.87 ± 98.50 | <0.001 |
| KC | 556.53 ± 121.08 | 30212.64 ± 109.51 | 28136.57 ± 2005.17 | 30600.09 ± 84.61 | <0.001 |
| LIF | 0.95 ± 0.46 | 60.67 ± 16.62 | 31.58 ± 6.64 | 377.38 ± 66.71 | <0.001 |
| G-CSF | 1159.18 ± 289.34 | 39854.47 ± 224.65 | 39942.15 ± 207.13 | 39855.60 ± 335.01 | <0.001 |
| GM-CSF | 46.33 ± 5.53 | 212.57 ± 19.99 | 92.79 ± 19.11 | 349.34 ± 29.02 | <0.001 |
| MCP-1 | 34.51 ± 6.32 | 3568.44 ± 1114.53 | 1182.31 ± 85.60 | 27602.38 ± 806.52 | <0.001 |
| RANTES (CCL5) | 17.96 ± 4.15 | 106.74 ± 14.70 | 108.34 ± 19.58 | 1321.31 ± 147.91 | <0.001 |
| VEGF | 2.67 ± 1.14 | 5.01 ± 0.81 | 13.75 ± 12.11 | 5.24 ± 2.19 | ns |

Mean concentrations ± SEM are given in pg/mL (*n* = 12 for sham mice; *n* = 3 in the 3h, 12h, and 24h FIP groups respectively; *ns*: not significant; *ND*: not determined).
